# Ensuring Quality Transitions From Early Years’ Establishments Into Primary Schools: Putting Research Into Practice

**DOI:** 10.3389/fpsyg.2021.499917

**Published:** 2021-09-21

**Authors:** Taryn Moir, Jayne Johnson

**Affiliations:** Inverclyde Educational Psychology Services, Greenock, United Kingdom

**Keywords:** preschool, transition, policy, early years, elementary school

## Abstract

Preschool to primary school transitions can be a time of anxiety for pupils, parents, and practitioners. The purpose of this study was to investigate what should be in place to support transitions and develop a shared understanding of what constitutes a nurturing transition for children. It is hoped that sharing this across service providers will increase consistency of practice. The design took a flexible qualitative approach to ensure the co-creation of shared meaning. Following an initial exploratory activity using a Person Centered Planning (PCP) approach, four qualitative activities were identified. Early years’ staff, primary teachers, parents, and pupils were recruited as participants from within a Scottish local authority to take part in these collaborative activities with qualitative and quantitative components, which enabled the co-construction of a transition timeline that articulates the core activities needed for optimal early years’ centers (EYC) to primary transitions. This timeline outlines various preparatory activities that can be taken for all pupils and also those with identified additional support needs (ASNs). In addition, examples of excellence were identified. While this document illustrates examples of good practice, it is not meant to replace other existing positive transition work; rather it is a guide that can enhance existing procedures within any given context.

## Introduction

Transitions and changes to the day of a child can be anxiety provoking and overwhelming (Geddes, 2006, p56) as individuals can struggle with taking on the new identities these changes require (Beach, 1999). If practitioners are not aware of these anxieties, they are likely to increase (Parent et al., 2018). Therefore, we need to plan for them appropriately (Bombèr, 2007, p114). Supporting transitions is described as one of the six nurture principles, which underpin relationship-based practice (March and Moir, 2018). These practices acknowledge that properly implemented, universal, mental wellbeing interventions improve academic achievement by 11% (Durlak et al., 2011).

Successful preschool transitions rely upon “activities and events that are designed to overcome the discontinuities that may disrupt children’s learning and development” (Love et al., 1992). Practitioners within the early years’ centers (EYC), the receiving primary school, and the broader multiagency team, such as educational psychologists, have a responsibility to ensure smooth transitions. Professionals working collaboratively maximize the opportunities to get transitions from early years to school right for children (Dockett, 2018). This is further enhanced when everyone working together and enabling involvement with parents, children and families (Pianta et al., 1999; Brooker, 2008; Robinson and Dunsmuir, 2010; Costales and Anderson, 2018).

## The Local Context

Within Scotland, a great deal of legislation guides early years’ practice (Education Scotland, 2010). Children are regarded as unique, and there is the need for enabling learning environments (Scottish Government, 2008). Furthermore, transitions matter (Education Scotland, 2010; Scottish Government, 2019) and these need to be effectively evaluated (Education Scotland, 2016).

Within Scotland, there is increased recognition of the potential impact of partnership working, and this is exemplified in the newly formed regional collaboratives, which aim to “drive improvements.” Each collaborative has various work streams. Within the Glasgow City Regional Education Improvement Collaborative (GCREIC) Achievement Framework Work stream, four sub-groups were convened, one being to develop principles for the transition process for the early years’ setting. In Scotland by the end of 2020, there will be an increase in the hours of free early years’ childcare to 1,140 h per year for all 3- and 4-year olds and eligible 2-year olds. This has led to the hypothesis that this increase in hours is likely to increase the number of between establishment transitions, which a child will face (Care Inspectorate, 2016). The Scottish Government recognizes the importance of quality support for transitions through early years’ settings and into school as this ensures continuity and progress (Scottish Government, 2016). In-line with the Scottish Government, the GCREIC Early Years Transition sub-group recognized that as the early years’ expansion is rolled out, there will be an increase in the number of transitions that children will be faced with. Local authority feedback has indicated that supporting transitions in the early years’ setting was an area of improvement, and it was recognized that the quality of transition and transition activities varied considerably within the authority (IEPS, 2018).

Local data highlighted that there are opportunities for Educational Psychologists to enhance the levels of partnerships with EYCs and other relevant partners (IEPS, 2018). Areas of work, which were considered useful, included action research, more training, more measuring of impact/research and evaluation, more sharing good practice/research, support with 1,140 h, more consistency of approach, implementation support, more evidence-based approaches and better consultation. Therefore, it was fitting that educational psychologists led in the EYC to primary school transition work.

Cross et al. (2006) noted that self-identity of professionals is often established in terms of their attitudes and beliefs, therefore, it was particularly important to explicitly recognize the experience that staff brought with them (Alsop, 2000). This is vital “if in an educational situation an adult’s experience is ignored. it is not just the experience that is being rejected, it is the person” (cited Cross et al., p71). Therefore, to maximize implementation success, practitioners needed to be fully involved in the process.

The project took the form of a two-part study. Stage 1 informed stage 2 by broadly investigating what constitutes optimum transitions. Stage 2 will then be concerned with how this can be implemented in practice.

## Aim

• To investigate what should be in place to support transitions.
• To develop a shared understanding of what makes a nurturing transition for children across providers.
• To create a document outlining the values and principles underpinning transitions for children.

## Stage 1

### Methods

#### Ethics

Ethical procedures of the local authority were adhered to throughout this study. The data obtained were screened for personal details and anonymised as necessary. Participants were made aware of their rights to withdraw at any time and debriefing took place for all.

#### Design

The overall design was a qualitative and flexible approach that took an interactive nature to co-create shared meaning of transitions. The initial stage 1 served as an exploration regarding the next steps.

#### Participants

Methods of recruitment for the investigation are consistent with obtaining active and informed consent from participants. The session was attended by 12 practitioners that included a mixture of early years’ staff, primary 1 teachers and deputes responsible for the early years’ transition process within an authority within the West Coast of Scotland.

#### Materials

**FIGURE 1:**
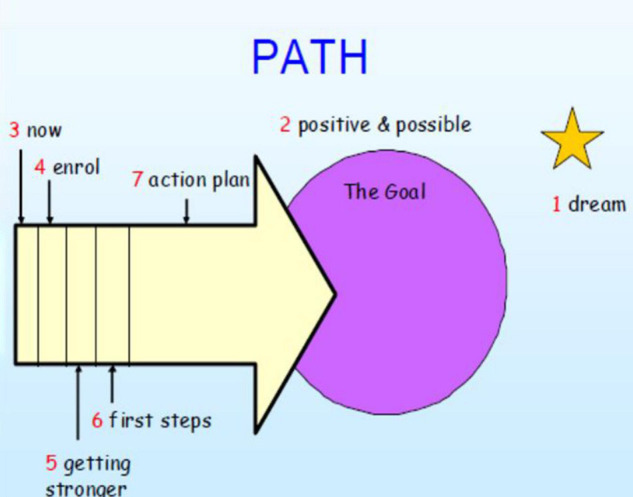
PATH process.

A large person centered planning (PCP) PATH was drawn onto a five connected pieces of flip chart paper, which is shown in Figure 1. Colored pens and post-it notes were provided for practitioners to use to contribute to the PATH. A PowerPoint was used to guide the session through each of the phases of the process.

#### Procedure

To optimize practitioner’s meaningful use of new transition guidelines, it was decided that they should be part of the process (Alsop, 2000). Therefore, the key features of the process to develop principles for the transition process were:

• Working together—all centers had developed transitional guidance unique to them.
• Consistency of practice could be ameliorated by working from the same guidance.
• Listening carefully to identify people aspirations for positive transitions.
• Identifying strengths and solutions.
• Focusing on what could be possible rather than just what was available. Local authority resources fluctuate yet the focus needed to be upon the creativity of resources.

Person Centered Planning is a creative and empowering method of planning and problem solving and can be used for organizing meetings (Robertson et al., 2005; Philip and McGregor, 2006). It is regarded as an adaptable tool for facilitating change in organizations. This approach was taken as it is based upon the principles of:

• Empowerment.
• The person or organization, their gifts and aspirations at the center of the planning process.
• Person-centered rather than resource-driven planning.
• Commitment to change and creative solutions.
• Promoting inclusion.

And its key features are:

• Working together.
• Listening carefully to identify what is important for people and what their aspirations are.
• Identifying strengths and solutions.
• Focusing on what could be possible rather than just what is available.
• Collaboratively developing an immediate and accessible visual plan, using graphics, images and words.

Therefore this process was chosen to enable practitioners to work collaboratively to develop an immediate and accessible visual plan, using graphics, images and words taking the PCP format as it is deemed both efficacious and effective in improving participation in choices (Robertson et al., 2005; Philip and McGregor, 2006).

The session goes through different phases to:

• Identify the dream asks questions like “What is your vision of the future regarding quality transitions for our children?” and “What are your best hopes for children’s transitions?”
• Identify goals (which should be positive and possible.
• Establish where we are now.
• Identify who can help us achieve the goals.
• Identify what is needed to keep going.
• Action plan.

#### Data Analysis

The whole group did the analysis of the data. Together, the group discussed the data and together themed it, ensuring the agreement of all the participants.

### Outcomes

The process was timed to ensure that each section had sufficient time for discussion. The arising themes from the PCP for early years’ transitions activity can be seen in Table 1 below. The aims of stage 2 were therefore identified as being:

**TABLE 1:** PCP themes.

**Dream Phase** Reoccurring themes (mentioned 3 + times) were coded in green	• Play based approaches in P1 to support transition and early literacy and numeracy development • ALL children have a smooth transition regardless of school A • coherent early level where prior learning is taken into account or and shared with establishment • Staggered admissions • Transition should reflect locality • Partnership working with health- more joined up • **Home-link and Barnardo’s and linking with other key agencies to get to know families before going to school Transition timeline which is longer than just January (often** • **waiting on placing requests)**	• Families familiar with school staff before starting school • **2 way visit learners/staff with protected time** • Relaxed confident happy parents. Happy to move forward. • **Professional dialog** • Trust • Equity • Clear communication • Progression in learning • Reduced waiting time for ASN to know school attending • Staff that understand the importance of relationship • Steps to school with CLD • Happy secure children
**Goal phase** (if red could be an action)	• Enhanced transitions meetings for children with ASN. Primary CTs included in school TAC/Girfec meetings • Parents and P1 teachers in nursery more prior to transition	• P1 children and staff visiting nursery playroom Term 1 and 2 to share pedagogy and observe Timeline for cluster transition
**Where are we now phase** (if red and area for development)	• Invitations to parents to visit classes • **Ensure all children get the same transition regardless of chosen school** • Current processes set up • **Timeline for transitions** • **Literacy focus in cluster could be developed beyond cluster** • **Sharing classes with nursery** • **Little evidence of school PEF being used for transitions** • **(early intervention)**	• **Make parents more aware of transition timeline** • **Social narratives to ensure a more positive transition** • School staff included in GIRFEC meetings • CLD first steps to school • Induction days • Using other parents’ experiences to ease parents’ anxieties e.g., • those with Jan/Feb birthday
**Enroll phase**	• Third sector • Community Learning and development • Home link- family support	• Parents • Private nursery’s • Health and Wellbeing coaches
**Getting stronger phase**	• Developing relationships • Professional trust	• Respecting professional judgment
**Action Plan phase**	• Forward plan- clearer timeline for transitions	• Look at pilot planning material- invitation to current group and include P1

• Develop a consistent timeline for transitions with multiagency partners (with additional guidance for children with additional needs).
• Ensure that parents’ experiences of early years’ to primary 1 transitions are gathered and their views regarding process improvements.
• Ensure that experiences of pupils of early years’ to primary 1 transitions are gathered.

Therefore the tasks for stage 2 as identified in stage 1 were:

• Invite early years and primary 1 teachers to form a subgroup to develop a transition locality timeline for all pupils and how this might be different for children with identified additional support needs (ASNs).
• Link with schools to negotiate how best to link with parents and invite them to take part in a semi-structured interview on their early years’ to primary 1 transition experience and what would make this better.
• Link with schools to negotiate how best to link with Primary 1 pupils and invite them to take part in an activity to express their early years’ to primary 1 transition experience.

### Discussion and Next Steps

The PCP activity had successfully identified some next steps. There was a keenness by EYC staff to continue to work collaboratively to enhance guidance on the transition process. The activity also showed that relationships between professionals were good; however, there was an ongoing drive to make this better.

Given that children with ASNs have varied and diverse needs; there was much debate about what was meant by an enhanced transition and was its process something that could be pre-prescribed. Complex needs add to the variety of psychological and psychosocial forces that underlie the behavior of a child, feelings, and emotions and thereby necessitates multidimensional understandings of the individual to determine what interventions are best within their plan (Mannino et al., 2017). What constitutes an additional need is defined in the Education (Additional Support for Learning) (Scotland) Act 2004 and 2009 thus “for whatever reason the child or young person is, or is likely to be, unable without the provision of additional support to benefit from school education provided or to be provided for the child or young person.” Furthermore, these needs can be long or short term. Examples of ASNs include children with physical or sensory issues, those being bullied or young carers. It may also include children affected by social difficulties or adverse family circumstances (e.g., Mannino and Schiera, 2017). Needs are diverse and may be multidimensional and complex. For example, for children who were migrants or refugees, a transition might involve learning to study in a new language, living in a new country and studying in a new educational system (Gunasekera et al., 2014). Yet, it is important to note that it is the **impact** of these factors, not their existence themselves that determine whether ASNs exist. Indeed, an ASN does not come in a vacuum and may have positive echo-systems ameliorating the need. For example, a child can have poor reading comprehension, yet has a highly skilled and attuned teacher who can mitigate the impact of these difficulties (Moir et al., 2020). Alternatively, a child may have low self-efficacy and self-esteem is given opportunities for outdoor physical fitness, which ameliorates their wellbeing, thereby improving their perception of self (Mannino et al., 2019). Therefore, it is crucial that all partners are involved in ongoing contextual assessment of the barriers of a child to learning and the necessary additional supports required.

As parental anxiety regarding transition is a predictor of child stress during the time it was considered important to include the views of their experience of transition (Parent et al., 2018) and their voice needed to be meaningfully captured. Similarly, in recent years there has been a growing recognition that the views of children on the early years’ to the primary process should also be acknowledged (Einarsdóttir, 2007). Obviously, transitions should be managed sensitively and building relationships with those who support the transition from the early stages facilitate smooth transitions and optimum engagement with both the parent and the child (Arnold and Baker, 2012).

Evaluation of the session was guided more by the levels of the discussion throughout the session than by evaluation form, yet this may have been a missed opportunity. However, the PCP format with participants taking an active role in answering questions indicated that participants had engaged well in the process. It had succeeded in the goal of informing the next steps regarding early years’ transitions. Throughout the discussion, it was evident that participants relished the opportunity to self-assess their practice and share best practice.

## Stage 2

### Aim

• To involve practitioners, parents and pupils in the development of an early years’ transition timeline for all pupils.

### Methods

#### Design

The overall design was a qualitative and flexible approach. The flexible design was chosen to allow for the co-creation of meaning to be formed through collaboration. The qualitative approach was taken to enable theoretical flexibility, which would allow for a variety of research questions (Braun and Clarke, 2006). It was considered useful in taking the key messages from a large amount of complex data (Guest et al., 2012). It also allows researchers to expand upon their lived experiences of transitions. Furthermore, this approach is generally useful in investigations of facilitators and barriers toward successful implementation (Guest et al., 2012). However, the evidence-based theory was referred throughout to increase external validity, while rival explanations were sought to increase internal validity. Informed by stages 1 and 2 which included:

a) A qualitative activity with practitioners to form the foundation timeline draft.
b) An evaluation process of early years’ to primary 1 transitions with parents.
c) An evaluation process of early years’ to primary 1 transitions with pupils.
d) A final activity to finalize the timeline with practitioners taking into account parents’ and pupils’ views.

#### Participants

Methods of recruitment for the investigation are consistent with obtaining active, informed consent from participants. Participants for tasks (a) and (d) were a mixture of early years’ staff, primary 1 teachers and deputes responsible for the early years’ transition process within an authority within the West Coast of Scotland. Although all practitioners from stage 1 were invited to take part and eight practitioners took part in (a) and four for (d).

Participants for the task (b) were opportunistic samples of 16 parents/carers or grandparents who were attending two schools to find out more about how their children were taught numeracy in school within the same authority on the West Coast of Scotland.

Participants for the task (c) were opportunistic samples of nine Primary 1 mixed ability children who had been selected with the support of a depute taking part in tasks (a) and (d). This was negotiated and agreed by the group at task (a). Ethical considerations including consent were taken as per stage 1.

### Task (a): Professionals Draft Timeline

#### Materials

A large timeline was drawn onto five connected pieces of flip chart paper indicating the school terms before and after the August transition point from early years to primary. Colored pens and post-it notes were provided for practitioners to use to contribute to the timeline. The PCP data from stage 1 and examples of other transition guidance were provided as the stimulus.

#### Procedure

Practitioners were asked to write on post-it notes all the transition activities that took place within their establishment, or that they had heard of, which would be considered best practice. There was then discussion to decide when each of these activities should take place to have optimum impact. The consensus was reached for each activity.

### Outcomes

The activity created a draft transition timeline.

### Task (b): Parental Interviews

#### Materials

Pens and paper.

#### Procedure

Parents took part in semi-structured interviews to ascertain how well they perceived the transition of their child into primary 1 from EYCs. They were asked why they thought it had gone well and what could have made it better.

#### Data Analysis

Quantitative rating scales were quantified, and a thematic analysis was undertaken to identify patterns of themes in qualitative interview data.

#### Results

Hundred percent said that the transition was very good or excellent. This was attributed to:

• Relationships.
  ∘ Knowing the children in the class.
  ∘ Friendships.
  ∘ Knowing key staff in the building (DHT/HT).
  ∘ Key staff taking the time to talk to children individually.
  ∘ Having siblings in the school.
• Child’s temperament.
• Good mix of children.
• Regular attendance at nursery.
• Buddy system.
• Going to school including PE time.

Areas for improvement included:

• Not having the half day initially as this was regarded as an additional transition into full-time school.
• Time in the actual classroom (rather than just PE/dinner hall).
• More visits (up to two afternoons per week from the preceding January).
• More information was provided to parents regarding their role in talking about and being excited about going to school.
• Parents and children going to visit the school together before any other transition activity.

### Task (c): Pupil Consultation

#### Materials

Coloring pens, crayons, pencils, and paper.

#### Procedure

Children were given the task of drawing their first day of school to prompt their memory regarding the transition. They were then asked to talk through their picture and talk about how that made them feel (Shorthouse and Shorthouse, 2018).

#### Results

All children reported feeling excited about their first day because:

• They enjoyed seeing their friends again.
• They liked their families being there to see them in school.

They also reported that:

• They had been to the school on visits.
• They had met their teachers.

Two children also reported that they were feeling shy, however, were unsure why this was.

### Task (d): Developing an Early Years’ Transition Timeline—Final Draft

#### Materials

The draft copy of the timeline. Pens, paper and post-it notes.

#### Procedure

Participants were asked to go through the draft timeline and decide by consensus what should be regarded as a core activity and what could be regarded as best practice.

#### Outcomes

Table 2 shows the core activities for either all children or those with identified ASNs.

**TABLE 2:** Core activities needed for transition from preschool to primary settings.

**An individualized approach taken throughout**		
**Time scale**	**Universal**	**Additional activities for ASN**
**Preschool year**		
August		Start arranging transition planning meetings
October-November	Information session to reassure parents regarding the transition process from Early Years to Primary School.	Meeting followed by observation by school staff within the EY context, followed by planning meeting outlining specific activities (additional visits etc.).
January- Easter	Enrollment at school	
	Getting to know you evening for parents (straight after enrollment deemed most appropriate time as this ameliorates parental anxieties)	
	Plan for Early Level continuity e.g., see book topic example of good practice	
	Children attend Gym sessions and school walk around including playground at PS	
Easter-May	Class teacher (if known or DHT if not known) visits nursery and has follow up meeting with staff. Class list is organized through discussions with EYCs as knowledge of the children helps create positive within class dynamics.	Meeting takes place to: • plan transition details • Consider which staff can develop good relationships with the children and allow time for them to do this Consider how to use the primary environment to support transition (e.g., access to play/nursery/nurture etc.)
	Invite pre-schoolers to school on days that classrooms are empty (P7s away)	Parents involved in walk around of school setting (afterschool) if beneficial
	Where possible make specific arrangements for children with agreed placing requests and ensure an equally rich transition process.	
	2 induction days • Children meet the buddies who will support them in P1 pupils in playground and dinner hall • Lunch trial with parents- an opportunity to speak to kitchen staff about menus and cashless cafeteria	
	P6/7 buddies identified and matched to preschool children	
June	Complete transition paperwork and share with school and parents	
**Year of starting school**		
August	SMT collate information from home and nursery for P1 teachers. Share along with ASN/CP files	
	In-service- SMT takes the opportunity for early level to discuss profiles/targets, GIRFEC pathways for new P1 children with P1 teachers	
	Early level E’s and O’s trackers/transition reports, SEAL planners/writing moderation skills checklists are transferred from EYC to school for P1 teachers to continue with	
P1’s first day (Aug)	Parents are invited to bring P1 children into the class room	
	Children meet their buddies who will support them in P1 pupils in playground and dinner hall	
October	Review transition process	

Examples of excellent practice were cited as follows:

“For children with ASN, after the initial TAC meeting in August, the school staff undertakes an observation within the EY context. This is followed by planning meeting outlining specific activities (additional visits etc.)”

“Within Inverclyde, it is standard to support transitions using book topic. This was considered especially useful because some schools have children coming from various establishments and all children knowing the same book within their EY experience would give them something else in common to discuss within primary 1. In August, EY and Primary staff have our first meeting to start planning for a book topic. Then in October, EY and Primary staff meet again to finalize the book selection and jointly consider the learning that can be drawn from it. We meet a third time, after Easter, to jointly discuss how the children have responded to the book topic and identify any learning themes that have been identified as needing additional learning opportunities within P1. In P1, the book and its characters can be revisited, perhaps using books within the same series to extend children’s learning. It is a 3-year programme allowing coverage of books related to Health and Wellbeing, Literacy and Numeracy. Books used to date have included Elmer (H&W) Shark in the Park (for literacy) and One Ted fell out of Bed for numeracy. Rotation of the book choices ensures that if a child is deferred they are not doing the same story twice. A moderation meeting of the book topic takes place in September once the children are in P1 to evaluate the process.

“From January to Easter, we advertise for P6 Nursery Ambassadors. Once selected they come to the nursery to learn about the children before being supported in the training of the rest of the P6 children who are going to become P1 buddies after the summer.”

“Buddies are involved in the Book bug programme and also do “snack on the go” so that children can learn to eat and play at the same time.”

“Where possible the early years’ children attend an assembly within the primary school at some point before they start school.”

“The Getting to know you, evening for parents happens straight after enrollment deemed most appropriate time as this may reduce parental anxieties. Where possible a crèche is made available.”

“A Play buddy from current P1 may be identified in June for some/all children; with particular thought to those who have additional support needs.”

“Photo books of staff and school are given/sent to parents before the summer holidays with a countdown calendar.”

“Attempts are made for EYECO’s from the various nursery’s visit P1 and support first morning. Parents sometimes go into the class to support the transition into Primary school on the first day if there are no EY staff available. On a few cases with children with additional support needs, this may continue for a few days but no longer than necessary/feasible.”

“From August, there are weekly P1 team meetings to discuss the planning and progress, pastoral care and support/challenge needed for the new P1 children.”

“From the August when the children are in P1, children are given the option of accessing the nursery for one afternoon per week until Christmas P1.”

“In the September after the children have started in P1, there is a Cluster meeting to discuss future transition planning for the year and sharing good practice.”

### Discussion

The aims of the study were met as an investigation took place into supporting EYC to primary transitions. Furthermore, the group developed a shared understanding of what a nurturing transition should look like for all children and those with ASNs. In addition, examples of good practice were collated to be shared. This informed the creation of a document that outlined the values and principles underpinning positive transitions for children. While there are many examples of transitions’ good practice within this document, it is not meant to replace other existing positive transition work, rather it is here to guide and enhance whatever exists within a given context.

Consistent with Love et al. (1992), practitioners were aware of the importance of preparative activities to aid successful transitions and were mindful of how successful planning could reduce both parental and pupil anxieties. The change in landscape within Scotland regarding the increased free childcare for early years’ children has enhanced the discussions between practitioners as they share their desire to get things right for children (Coles et al., 2016). This landscape provides increased opportunities for positive outcomes for children but is not without its difficulties (Scottish Government, 2016), and consistency of quality outcomes is an ongoing goal.

One barrier to the consistency of practice is that while many children transitioned from the EYC to the same school, other children would move to a different school. Due to time pressures and more remote working relationships with relevant personnel, these transitions are vulnerable to lesser quality planning and are still an area for development.

There is still further research trying to ascertain which transition activities have the greatest impact. In addition, what is the impact on the early years’ practitioner and/or primary 1 teacher when transitions do and do not go to plan.

The study had several limitations. Parental involvement was opportunistic and therefore may be subject to bias as not representative of the population. Larger pools of samples may have further refined the timeline and guidance. In addition, the activities that the PCP informed were done with separate groups of parents, children or practitioners. There may have been greater synergy and higher quality collaborations if all stakeholders took part in the same activities at the same time. In retrospect having children and parents involved in stage 2 was not ideal, perhaps having them involved from the initial conception of the project, as major stakeholders, would have offered a more authentic timeline.

In addition, this study took a flexible design, which is a radical departure from the statistical sampling paradigm, (Robson, 1995). However, this was deemed necessary to ensure that a co-creation of meaning could be achieved, and this involvement of all participants through the process would ensure greater readiness by partners to implement these guidelines (Moir, 2018).

Furthermore, the use of the qualitative approach was used to encourage practitioners to expand their responses and express the depth of feeling about transitions, which could be used as rich data to inform the guidance. However, different researcher interpretations can reduce the reliability of data (Guest et al., 2012) and due to the flexibility of qualitative approach, there can be a variety of different ways to focus on the data. Therefore, one of the limitations of the study is the lack of quantitative data. Indeed, a multiple methods approach, which combined the rationalistic quantitative with the naturalistic qualitative information, would have increased levels of construct validity.

### Next Steps: Implementation and Evaluation Plan

Viennet and Pont (2017) report that there are significant challenges when translating policy into everyday practice. To ensure effective implementation, it is necessary to share the timeline with all local authority educational psychologists and senior education staff and facilitating professional discussions to ensure it is fully embedded (Moir, 2018). Representatives from early years and primary schools will then be invited to form an Implementation Team. The Implementation Team will share the use of the timeline with key staff in their educational establishment and have the responsibility of ensuring staff considers the recommendations when planning the transitions for children. Thereafter, they will monitor and evaluate how effectively staff follow the timeline and highlighting practices that are either “over and above” the recommendations or any barriers that arise. From this information, the guidance can be reviewed and updated as appropriate.

## Implications for Wider Research

This research reinforces the importance of starting the transition process earlier, while still maintaining high expectations of success (Francis et al., 2018). Similarly, it was recognized that while all need opportunities to get used to the new school context, some children with ASNs, will require more support, and this relies upon good communication between the EYC, school, home and any other partner agencies which may be involved (Nuske et al., 2019). There are many parallels between themes of transitions in the early years’ sector and other forms of transition. For example, generally, transitions are about dealing with uncertainty and therefore preparation brings about optimum outcomes (Formica et al., 2017). Furthermore, transition outcomes of the children can be related to the levels of confidence that those managing the transition have (Santisi et al., 2018). In this case, early years and school staff appreciated the opportunity to be involved in creating guidance that ultimately increases their professional confidence and thereby increased their readiness to further support these transitions.

This study showed consistency with existing literature, which indicates that most children report that they look forward to going to school (Broström, 2019) and for optimum planning parents should be fully involved in the transition process (Graham, 2019). Indeed, there is agreement that there are role and responsibilities of all partners and stakeholders in the process, and this relies upon cooperative relationships (Einarsdottir et al., 2019) and good communication (Nuske et al., 2019). This is particularly important when considering children with diverse support needs.

## Conclusion

In conclusion, while it is recognized that all activities need to be undertaken collaboratively with partners and stakeholders; there are certain activities that are led by the EYC, and others are led by the receiving primary school or parent. Therefore, working together as a multiagency group or team around the child is essential to maximizing positive transitions (Pianta et al., 1999; Brooker, 2008; Robinson and Dunsmuir, 2010; Costales and Anderson, 2018).

The qualitative nature of this co-creative process ensured that stakeholders had a choice around what best practice and consistency would look like and therefore paralleled the involvement that all partners and stakeholders should have when specific child’s plans; thereby illustrating the value of an inclusive approach.

## Data Availability Statement

All datasets generated for this study are included in the manuscript/supplementary material.

## Ethics Statement

This study involves human participation and was therefore reviewed and approved by Inverclyde Educational Psychology Services Ethical Considerations group. The participants provided their written and verbal informed consent to participate in this study.

## Author Contributions

Both authors listed have made a substantial, direct and intellectual contribution to the work, and approved it for publication.

## Conflict of Interest

The authors declare that the research was conducted in the absence of any commercial or financial relationships that could be construed as a potential conflict of interest.

## Publisher’s Note

All claims expressed in this article are solely those of the authors and do not necessarily represent those of their affiliated organizations, or those of the publisher, the editors and the reviewers. Any product that may be evaluated in this article, or claim that may be made by its manufacturer, is not guaranteed or endorsed by the publisher.
